# Valor Prognóstico de Níveis Elevados de Troponina I Isolados em Pacientes sem Síndrome Coronariana Aguda Admitidos no Serviço de Emergência

**DOI:** 10.36660/abc.20190356

**Published:** 2021-05-06

**Authors:** Célia Domingues, Maria João Vidigal Ferreira, Joana Moura Ferreira, Ana Vera Marinho, Patrícia Marques Alves, Cátia Ferreira, Isabel Fonseca, Lino Gonçalves

**Affiliations:** 1 Universitário de Coimbra EPE Centro Hospitalar Coimbra Portugal Centro Hospitalar e Universitário de Coimbra EPE, Coimbra - Portugal; 2 Universidade de Coimbra Faculdade de Medicina Coimbra Portugal Universidade de Coimbra - Faculdade de Medicina, Coimbra - Portugal

**Keywords:** Troponina I, Prognóstico, Pronto-socorro, Lesão Miocárdica Não Isquêmica

## Abstract

**Fundamento:**

Embora a elevação não isquêmica da troponina seja frequentemente observada em pacientes admitidos no pronto-socorro (PS), não há consenso quanto ao seu manejo.

**Objetivos:**

Este estudo teve como objetivo caracterizar os pacientes admitidos no PS com elevação da troponina não-isquêmica e identificar potenciais preditores de mortalidade nessa população.

**Métodos:**

Este estudo observacional retrospectivo incluiu pacientes do PS com resultado positivo no teste da troponina entre junho e julho de 2015. Pacientes com diagnóstico clínico de síndrome coronariana aguda (SCA) foram excluídos. Os dados demográficos dos pacientes e as variáveis clínicas e laboratoriais foram extraídos dos prontuários médicos. Os dados do seguimento foram obtidos por 16 meses ou até a ocorrência de morte. O nível de significância estatística foi de 5%.

**Resultados:**

A elevação da troponina sem SCA foi encontrada em 153 pacientes no PS. A mediana (IIQ) de idade dos pacientes foi de 78 (19) anos, 80 (52,3%) eram do sexo feminino e 59 (38,6%) morreram durante o seguimento. A mediana do período de seguimento (IIQ) foi de 477 (316) dias. Os sobreviventes eram significativamente mais jovens 76 (24) vs. 84 (13) anos; p=0,004) e apresentaram uma maior proporção de elevação da troponina isolada (sem elevação da creatina quinase ou mioglobina) em duas avaliações consecutivas: 48 (53,9%) vs. 8 (17,4%), p<0,001. Os sobreviventes também apresentaram menor taxa de tratamento antiplaquetário e internação no mesmo dia. Na regressão logística multivariada com ajuste para variáveis significativas na análise univariada, a elevação isolada da troponina em duas avaliações consecutivas mostrou *hazard ratio* = 0,43 (IC95% 0,17–0,96, p=0,039); hospitalização, tratamento antiplaquetário anterior e idade permaneceram independentemente associados à mortalidade.

**Conclusões:**

A elevação isolada da troponina em duas medidas consecutivas foi um forte preditor de sobrevida em pacientes no PS com elevação da troponina, mas sem SCA.

## Introdução

O infarto do miocárdio (IM) clínico, de acordo com a quarta definição universal, requer a presença de lesão miocárdica aguda detectada por biomarcadores cardíacos anormais associados a evidências de isquemia miocárdica aguda. A troponina cardíaca (Tnc) acima do ponto de corte do percentil 99, com padrão crescente ou decrescente, é o biomarcador de lesão miocárdica,^1^ porque não pode ser liberada por tecidos não cardíacos e tem excelente acurácia para o diagnóstico de infarto agudo do miocárdio.^1^^–^^3^

A Tnc é uma proteína distribuída no citoplasma e sarcômero de um miócito cardíaco, principalmente no retículo sarcoplasmático. Três subunidades constituem o complexo da troponina, um componente inibitório (troponina I), componente de ligação à tropomiosina (troponina T) e componente de ligação de cálcio (troponina C).^4^

As subunidades T e I (TncT e TncI, respectivamente) são específicas do músculo cardíaco e, portanto, podem atuar como marcadores adequados de lesão cardíaca. A TncT apresenta uma descarga dupla, primeiro o componente citoplasmático e depois o componente de ligação.^5^ A TncI é específica para o coração, e não foi identificada no músculo esquelético. Esta especificidade de 100% mostra que a TncI pode ser um marcador de necrose miocárdica (MNM) ideal.^6^

Antes do advento da troponina, os MNM anteriores utilizados eram a creatina quinase-isoenzima muscular/cerebral (CK-MB) e a mioglobina, que eram menos sensíveis e não específicas para IM.^7^^,^^8^ Por essa falta de sensibilidade e especificidade, elas foram progressivamente excluídas das investigações de CSA.^2^^,^^3^

Embora as subunidades Tnc sejam fortemente específicas para miócitos cardíacos, elas podem ser liberadas sob um amplo espectro de condições patológicas não cardíacas, como sepse, doença renal crônica, emergências hipertensivas, sangramento gastrointestinal, acidente vascular cerebral e rabdomiólise.^6^^,^^9^ Nesse cenário, a detecção da troponina pode ser o resultado de 5–8% da liberação do componente citosólico em resposta ao *turnover* celular do miócito, liberação celular de produtos de degradação e aumento da permeabilidade da parede celular.^10^

O uso generalizado de ensaios de troponina no pronto-socorro (PS) pode representar um desafio diagnóstico difícil quando o teste é anormal em paciente sem SCA.^11^ De acordo com a literatura, níveis elevados de Tnc em pacientes sem SCA foram associados a um prognóstico ruim.^10^^,^^12^^–^^16^

## Objetivo

Este estudo teve como objetivo identificar os fatores preditivos de mortalidade/sobrevida em pacientes de PS sem SCA e TncI elevada utilizando as características do paciente, histórico clínico, comorbidades e valores analíticos (incluindo creatinina, CK-MB, mioglobina e TncI) medidos no PS.

## Métodos

### Desenho, local e participantes do estudo

Neste estudo retrospectivo, analisamos os dados laboratoriais de pacientes consecutivos que vieram ao PS de um hospital universitário da comunidade durante o período de 1 mês, de junho a julho de 2015, e selecionamos pacientes que apresentaram elevação da TncI.

Todas as informações clínicas foram coletadas, incluindo registros médicos e de enfermagem do PS e hospitalizações, análises e outros exames complementares. O seguimento foi realizado através de registros locais e nacionais para observar as taxas de reinternação cardiovascular e morte.

Os dados coletados incluíram dados demográficos, fatores de risco cardiovascular, ensaio de biomarcador miocárdico e resultados da creatinina, sintoma principal no PS, diagnóstico final do PS, hospitalização, mortalidade da hospitalização, taxa de mortalidade em 30 dias e 16 meses e taxa de reinternação cardiovascular.

Neste estudo, os critérios para o diagnóstico de infarto agudo do miocárdio foram: aumento ou diminuição da Troponina I com pelo menos um valor anormal acima do limite superior de referência do ensaio e, ao menos um dos seguintes: 1) sintomas de isquemia; 2) novas alterações da onda T / segmento ST ou novo bloqueio de ramo esquerdo; 3) desenvolvimento de ondas Q patológicas na eletrocardiografia; 4) nova perda de miocárdio viável ou anormalidades regionais de movimento da parede na imagem; ou 5) identificação de um trombo intracoronário na imagem.^1^

Os registros do PS e a hospitalização de todos os pacientes com TncI elevada foram revisados pelos investigadores, e os pacientes foram divididos em dois grupos: aqueles com diagnóstico de SCA (IM tipo 1 ou tipo 2 com sinais e sintomas isquêmicos (com vasoespasmo, embolia e dissecção coronária não-aterosclerótica) (grupo A) e aqueles sem SCA com um teste de troponina positivo devido ao desequilíbrio de oferta/demanda de oxigênio ou lesão miocárdica sem sinais ou sintomas de isquemia miocárdica aguda (grupo B). Pacientes com IM tipo 4 ou 5 não foram incluídos neste estudo, pois, por definição, estes não são os pacientes regulares do PS e os pacientes com IM tipo 3 não têm medida de TncI.^1^

Pacientes sem SCA foram identificados através de critérios predefinidos que incluíram o seguinte: 1) miocardite/cardiomiopatia: diagnóstico à alta hospitalar ou achados sugestivos de miocardite em teste de imagem ou patológico, cardiomiopatias infiltrativas, como amiloidose ou sarcoidose, uma fração de ejeção ≤30% antes da admissão, ou transplante cardíaco anterior; 2) infecções: condições com impacto sistêmico, como celulite, pneumonia, sepse e pielonefrite; 3) disritmias agudas não relacionadas com a SCA; 4) doença renal crônica ou aguda: doença renal crônica estágio 5, diálise crônica, receptor de transplante renal ou doença renal aguda moderada a grave; 5) patologia do sistema nervoso central: acidente vascular cerebral, convulsão ou hemorragia subaracnóidea; 6) sangramento abdominal ou gastrointestinal agudo; 7) embolia pulmonar; 8) síncope inexplicada; 9) asma ou exacerbação de doença pulmonar obstrutiva crônica; e 10) outros: nível elevado de troponina de etiologia desconhecida que não atende a nenhum dos critérios acima mencionados.

O *endpoint* primário para este estudo foi a mortalidade durante a hospitalização, em 30 dias e 16 meses, enquanto o *endpoint* secundário foi a reinternação por doença cardiovascular durante o seguimento.

O seguimento foi concluído em 16 meses ou em caso de morte. O seguimento foi realizado através de consulta aos prontuários eletrônicos e ao registro nacional de óbitos online. O conselho de revisão institucional aprovou o protocolo do estudo. A exigência de consentimento informado foi dispensada porque os pacientes não receberam qualquer tipo de cuidado diferente por causa do estudo.

### Ensaios de marcadores de necrose miocárdica

O nível de troponina I foi determinado utilizando o mesmo imunoensaio TncI padrão (Troponina I Siemens Dimension EXL)^17^^-^^19^ em todos os pacientes. O teste foi realizado no laboratório central do hospital. Os limites de detecção inferior e superior estabelecidos pelo fabricante foram 0,017 ng/mL e 4000 ng/mL, respectivamente. As medidas abaixo do limite de detecção receberam o valor de 0. Os resultados do teste de troponina I foram considerados positivos se o nível fosse superior ao limite de referência (> 0,059 ng/mL) usado no laboratório do PS. Os resultados dos ensaios de CK-MB e mioglobina foram considerados normais quando <3,6 ng/mL e 9–82 ng/mL, respectivamente.

Medidas repetidas dos MNM foram realizadas pelo menos 3 horas após a primeira avaliação.

Utilizando a primeira e a segunda avaliação da Tnc, a variação foi calculada da seguinte forma: variação da troponina% = ((segunda troponina × 100)/primeira troponina) × 100%.

### Métodos estatísticos

Todos os dados contínuos foram testados quanto à normalidade com o teste de Shapiro-Wilks; todos apresentaram distribuição não-normal e são apresentados por sua mediana e intervalo interquartil. O teste de Mann-Whitney foi aplicado para comparar variáveis contínuas. As variáveis categóricas foram representadas por sua frequência e comparadas utilizando o teste exato de Fisher ou o teste de qui-quadrado.

A sobrevida foi analisada utilizando modelos de risco proporcional de Cox uni- e multivariados. Os resultados foram expressos como *hazard ratio* (HR) com intervalos de confiança de 95% (IC 95%). Para as variáveis independentes preditoras de sobrevida, um gráfico de sobrevida foi obtido utilizando o método de Kaplan-Meier e o teste de *log-rank*. O nível de significância estatística foi estabelecido com um valor de p<0,05. Todas as análises estatísticas foram realizadas utilizando o software SPSS 23.0 para Mac (SPSS, Inc; Chicago, IL, EUA).

## Resultados

### Características basais e diagnósticos da população de estudo

**Figura 1:**
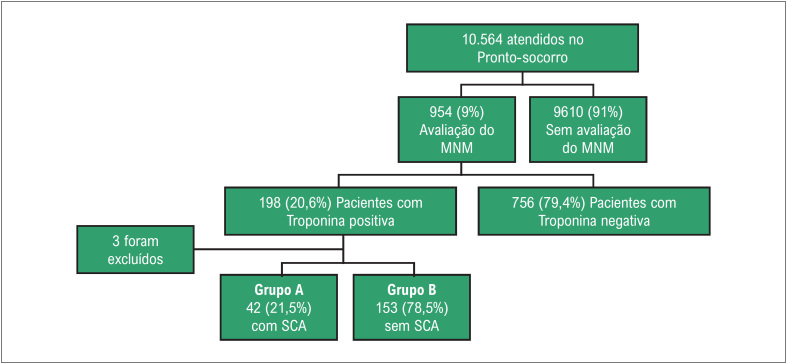
Ilustração esquemática dos pacientes incluídos. MNM, marcadores de necrose miocárdica; SCA, síndrome coronariana aguda

Durante o período de estudo de 1 mês, 10.564 pacientes foram admitidos no PS da nossa instituição. Os pacientes que foram submetidos a avaliações de MNM, incluindo troponina I, CK-MB e mioglobina e sua distribuição de acordo com o status da troponina e o diagnóstico final, estão descritos na Figura 1. Os pacientes foram divididos em dois grupos: Grupo A (n = 42 [21,5%]) com SCA (todos com oclusão/sub-oclusão aguda de artérias coronárias: 4 deles com IM tipo 2 (2 casos de dissecção coronária e 2 de trombose coronária embólica); de 38 com IM tipo 1, 14 apresentavam IAM com supradesnivelamento do segmento ST (IAMCSST); enquanto no Grupo B (n = 153 [78,5%]) sem SCA, 58 apresentavam desequilíbrio entre oferta/demanda de oxigênio, 53 tinham lesão miocárdica aguda sem sinais ou sintomas de isquemia e 42 tinham elevação estável da TncI (variação da TncI em duas análises consecutivas ≤ 20%). Entre os pacientes sem SCA, a primeira medida de MNM foi realizada após uma mediana de 6 (IIQ 4) horas desde o início dos sintomas, e 90 pacientes repetiram a medida de MNM após uma mediana de 5 (IIQ 3) horas a partir da primeira avaliação.

Na primeira avaliação do MNM do grupo B, 81 pacientes apresentaram elevação de CK-MB e/ou mioglobina (16 apresentaram elevação de CK-MB, e 40 de mioglobina, enquanto 25 tiveram elevação de ambos). Na segunda avaliação, 18 apresentaram elevação de ambos e 6 apresentaram elevação isolada do CK-MB e 31 da mioglobina. Em ambas as avaliações, 88 pacientes apresentaram pelo menos uma elevação de CKMB e / ou mioglobina.

**Figura 2:**
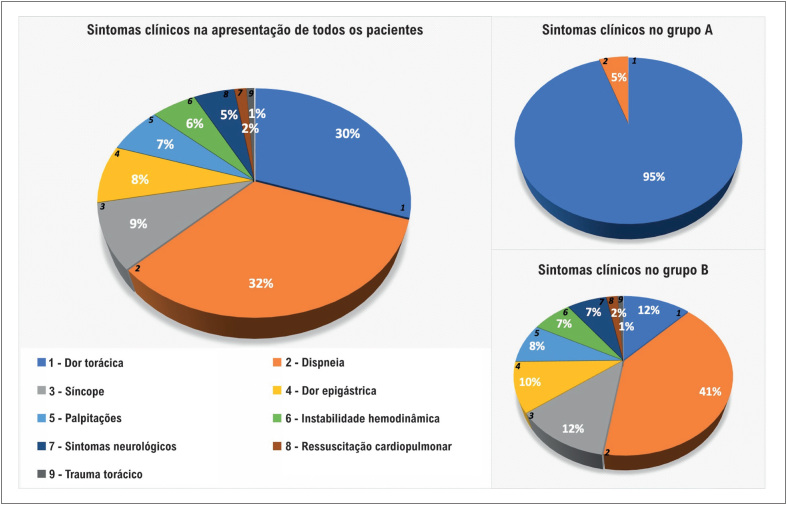
Ilustração esquemática dos sintomas clínicos na apresentação de todos os pacientes e diferentes grupos A: com SCA e grupo B: Sem SCA.

Os pacientes com resultado positivo no teste da troponina apresentaram um amplo espectro de sintomas clínicos na apresentação (Figura 2). Como esperado, os pacientes que ao final receberam o diagnóstico de SCA (Grupo A) apresentaram maior proporção de dor torácica como queixa principal na apresentação hospitalar.

Conforme mostrado na Tabela 1, os pacientes dos Grupos A e B possuíam mediana de idade semelhante, mas tinham proporções de gênero significativamente diferentes. Em relação aos fatores de risco cardiovascular e condições de comorbidade, não foram encontradas diferenças significativas na prevalência de diabetes mellitus e hipertensão, mas hiperlipidemia e doença arterial coronariana anterior foram mais comuns em pacientes com SCA e insuficiência cardíaca anterior e tratamento anticoagulante foram mais prevalentes em pacientes sem SCA.

**Tabela 1:** Características basais de todos os pacientes com elevação da troponina no PS

	Global N = 195	Grupo A n = 42 (22%)	Grupo B n = 153 (78%)	p-valor
Idade (anos), mediana (IIQ)	77(21) anos	71(19) anos	78(19) anos	0,06
Sexo masculino n (%)	105 (53,8%)	32 (76,2%)	73 (47,7%)	0,001
Diabetes mellitus, n (%)	69 (35,5%)	16 (37,2%)	54 (35%)	0,89
Hipertensão, n (%)	155 (79,3%)	35 (84,4%)	119 (78,1%)	0,57
Hiperlipidemia, n (%)	87 (44,4%)	25 (60%)	61 (40,1%)	0,03
DAC anterior, n (%)	37 (19%)	13 (31%)	24 (16%)	0,02
IC anterior, n (%)	43 (21,9%)	9 (21,9%)	71 (46,7%)	0,02
AVCI anterior, n (%)	31 (14,8%)	3 (6,3%)	26 (16,8%)	0,26
TFG (ml/[min.1.73 m^2^]), mediana (IIQ)	54 (46)	68 (47)	49(47)	0,10
**Medicação Anterior**				
Anticoagulantes	62 (33,7%)	4 (10%)	47 (31%)	0,007
Antiplaquetários	50 (26,7%)	15(37%)	48 (32%)	0,13
Betabloqueadores	69 (36,9%)	16 (40%)	53 (36,1%)	0,65
Inibidores ECA/ARA	108 (57,8%)	23 (57,5%)	87 (57,8%)	0,97
ARM	15 (17,6%)	2 (12,5%)	13 (18,8%)	0,55
Estatinas	83 (44,4%)	24 (60%)	60 (40,1%)	0,02
Troponina padronizada na primeira avaliação *, n (%)				<0,001
1–2,99	95 (48,9%)	10 (23,1%)	85 (55,8%)	
3-4,99	36 (18,3%)	5 (12,8%)	31 (19,7%)	
5-9,99	19 (9,7%)	4 (10,3%)	15 (9,7%)	
10+	45 (23,1%)	23 (53,8%)	22 (15%)	
CK-MB elevada, n (%)	54 (53%)	22 (53%)	32 (21%)	<0,001
Mioglobina elevada, n (%)	88 (48%)	23 (56%)	66 (45%)	0,24
CK-MB + Mioglobina elevadas, n (%)	43 (22%)	16 (40%)	25 (16%)	0,003
% de elevação da troponina entre 2 medidas, mediana (IIQ)	7 (73)	183 (666)	2,65(42)	<0,001

Grupo A - pacientes com síndrome coronariana aguda (SCA); Grupo B - pacientes sem SCA. DAC: doença arterial coronariana; IC: insuficiência cardíaca; AVCI: acidente vascular cerebral isquêmico agudo; TFG: taxa de filtração glomerular de acordo com a equação MDRD; Inibidor de ECA: inibidor da enzima conversora da angiotensina; ARM: antagonista do receptor mineralocorticoide.

Os principais diagnósticos dos pacientes do Grupo B foram miocardite /cardiomiopatia (40 [26%]), seguido de infecção (celulite, pneumonia e pielonefrite, 24 [15,5%]), arritmias agudas (25 [16,6%]), doença renal crônica ou aguda (17 [11%]), doença cerebral (13 [8,4%]), sangramento abdominal ou gastrointestinal agudo (11[7,1%]), embolia pulmonar (6 [3,9%]), síncope inexplicada (4 [2,6%]]), asma ou exacerbação de doença pulmonar obstrutiva crônica (4 [2,6%]) e outros (9 [6,5%]).

### Dados de desfechos

A mediana do seguimento (IIQ) foi de 477 dias (316). Não foram encontradas diferenças significativas entre os grupos em relação à mortalidade intra-hospitalar (6 [14,3%] vs. 21 [13,7%], p = 0,077), mortalidade em 30 dias (6 [14,3%] vs. 27 [17,6%], p = 0,4) e reinternação cardiovascular no seguimento (11 [29,7%] vs. 32 [24,2%], p = 0,316). Notavelmente, a taxa de mortalidade em longo prazo foi significativamente maior nos pacientes do grupo B (9 [21,4%] vs. 59 [38,6%], p = 0,039), embora as curvas de sobrevivência dos dois grupos não fossem significativamente diferentes (log rank, 3,45; p = 0,063). As principais causas de morte do grupo B foram: cardiovasculares em 12 indivíduos (nenhum deles com diagnóstico de infarto agudo do miocárdio), 27 não-cardiovasculares, 13 por causas desconhecidas e 4 mistas de causas cardiovasculares e outras comorbidades.

### Resultados principais

Os preditores de mortalidade dos pacientes do Grupo B estão descritos na Tabela 2. Foi observado que maior idade (p <0,001), insuficiência cardíaca anterior (p = 0,049), medicação antiplaquetária anterior (p = 0,005) e hospitalização após a avaliação índice do PS (p < 0,001) foram preditores de mortalidade. A morte no seguimento não foi relacionada com os níveis de TncI, CK-MB ou mioglobina (primeira, segunda ou ambas as avaliações). No entanto, a elevação isolada da troponina (isto é, sem elevação concomitante da CK-MB ou mioglobina) foi um poderoso preditor de sobrevivência; de fato, a elevação isolada da troponina na primeira medida estava presente em 58,7% dos sobreviventes vs. 40% dos não sobreviventes (p = 0,021); os pacientes com duas medidas isoladas de elevação da troponina tinham maior probabilidade de sobreviver (48 [53,9%] dos sobreviventes vs. 8 [17,4%] dos não sobreviventes [p <0,001]).

**Tabela 2:** Associação entre as Variáveis Clínicas e Sobrevida em Longo Prazo de Pacientes com Elevação da Troponina e Síndrome Coronariana Não-Aguda (Grupo B)

	Sobreviventes (n = 94)	Não-sobreviventes (n = 59)	p-valor
Idade, mediana (IIQ)	76(24) anos	84 (13)	<0,001
Sexo masculino, n (%)	44 (46,8%)	29 (49,2%)	0,77
**Fatores de risco CV, n (%)**			
Diabetes mellitus	28 (30,8%)	22 (38,6%)	0,33
Hipertensão	68 (73,9%)	44 (78,6%)	0,52
DAC anterior	14 (15,2%)	9 (16,4%)	0,85
HF anterior	33 (39,3%)	31 (56,4%)	0,049
TFG, mL / (min.1.73 m^2^), mediana (IIQ)	56 (48)	45 (34)	0,05
Frequência cardíaca, bpm, mediana (IIQ)	75 (33)	84 (36)	0,10
**Medicação Anterior, n (%)**			
Antiplaquetária	22 (23,9%)	25 (35,5%)	0,02
Anticoagulantes	34 (37%)	12 (21,8%)	0,06
Betabloqueadores	35 (38%)	18 (32,7%)	0,52
Inibidor de ECA	57 (62%)	28 (50,9%)	0,19
ARM	9 (20,5%)	4 (16%)	0,65
Estatinas	36 (39,1%)	23 (41,8%)	0,75
**ECG, n (%)** Sem alterações significativas	51 (64,6%)	16 (50%)	0,38
Elevação do ST	1 (1,3%)	0 (0%)	
Depressão do ST ou onda T negativa	16 (20,3%)	6 (18,8%)	
Fibrilação atrial	28 (35,0%)	18 (46,2%)	
BRE	4 (5,1%)	4 (12,5%)	
Ritmo	4 (5,1%)	3 (9,4%)	
**Marcadores de necrose miocárdica, mediana (IIQ)**			
Troponina (ng/mL) na primeira avaliação	0,13 (0,23)	0,10 (0,18)	0,61
CK-MB (ng/mL) na primeira avaliação	1,6 (1,8)	1,9 (2,05)	0,50
Mioglobina (ng/mL) na primeira avaliação	70 (120)	99 (175)	0,06
Elevação isolada da troponina na primeira avaliação, n (%)	54 (58,7%)	22 (40%)	0,028
Troponina (ng / mL) na segunda avaliação, mediana (IIQ)	0,12(0,16)	0,14(0,32)	0,28
% de variação da troponina I em duas medidas sequenciais, mediana (IIQ)	0 (32)	27 (35)	0,002
Elevação isolada da troponina em duas medidas sequenciais, n (%)	48 (53,9%)	8 (17,4%)	<0,001
Hospitalização no evento índice, n (%)	52 (55,3%)	51 (86,4%)	<0,001
Revascularização coronária			0,88
Sem terapia específica, n (%)	88 (93,6%)	54 (91,5%)	
TMO, n (%)	5 (5,3%)	4 (6,8%)	
ICP + TMO, n (%)	1 (1,1%)	1 (1,1%)	

**Figura 3:**
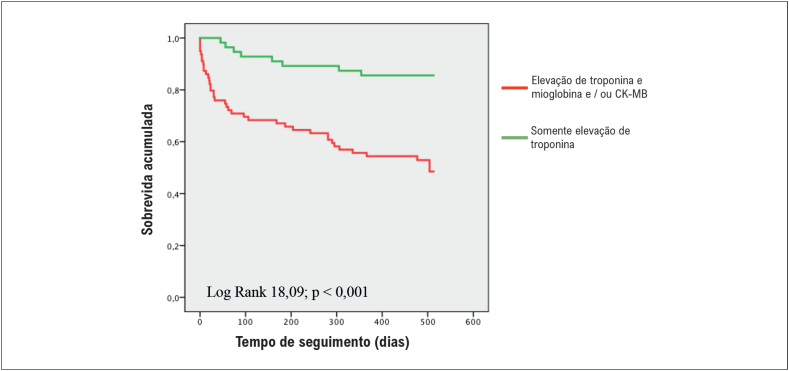
Curva de sobrevida de Kaplan-Meier no seguimento de 16 meses de pacientes não-SCA de acordo com resultados de duas avaliações consecutivas de MNM.

IIQ: intervalo interquartil; DAC: doença arterial coronariana; IC: insuficiência cardíaca; AVCI: acidente vascular cerebral isquêmico agudo; TFG: taxa de filtração glomerular de acordo com a equação MDRD; Inibidor da ECA: inibidor da enzima conversora da angiotensina; ARM: antagonista do receptor mineralocorticoide; ECG: eletrocardiograma; CK: creatina-quinase; CK-MB: creatina quinase-MB; TMO: terapia médica otimizada; ICP: intervenção coronária percutânea;

A análise de regressão de Cox é apresentada na Tabela 3. A análise univariada mostrou que em pacientes internados com troponina isolada em duas medidas consecutivas de MNM, a probabilidade de sobrevida em longo prazo no seguimento aumentou em quatro vezes (p <0,001). A análise multivariada de Cox corrigida para idade e sexo demonstrou que a elevação isolada da troponina em duas medidas consecutivas permaneceu um preditor independente de sobrevida (HR, 0,433; IC95%, 0,196-0,958; p = 0,039). A Figura 3 mostra as curvas de sobrevida dos pacientes do Grupo B de acordo com a presença de elevação isolada da troponina em duas medidas consecutivas (*log rank*, 18,09; p <0,001).

**Tabela 3:** Análise de regressão de Cox univariada e multivariada (corrigida para idade e sexo) das variáveis clínicas e sobrevida em longo prazo de pacientes com elevação da troponina e síndrome coronariana não-aguda

	Regressão univariada de Cox		Regressão multivariada de Cox	
	HR (IC95%)	p-valor	HR (IC95%)	p-valor
Idade, anos	1,040 (1,017–1,063)	0,001	1,030 (1,002–1,058)	0,038
Sexo	0,899 (0,540–1,499)	0,684	0,807 (0,436–1,493)	0,494
**Fatores de risco CV**				
Diabetes mellitus	1,336 (0,783–2,277)	0,288		
Hipertensão	1,245 (0,658–2,358)	0,501		
DAC anterior	1,067 (0,521–2,182)	0,860		
HF anterior	1,649 (0,967–2,812)	0,066		
GFR, mL / (min.1.73 m^2^)	0,992 (0,984–1,001)	0,082		
Frequência cardíaca, bpm	1,006 (0,997–1,014)	0,193		
**Medicação Anterior**				
Antiplaquetários	1,867 (1,230–2,835)	0,006	1,823 (1,105–3,006)	0,019
Betabloqueadores	0,806 (0,459–1,416)	0,449		
Inibidor de ECA	0,689 (0,406–1,170)	0,170		
ARM	0,764 (0,262–2,226)	0,611		
Estatinas	1,017 (0,595–1,739)	0,950		
Padrão de ECG	1,162 (0,999–1,351)	0,067		
**Laboratório**				
Elevação isolada da troponina na primeira avaliação	0,533 (0,311–0,916)	0,021	1,097 (0,378–3,180)	0,865
Elevação isolada da troponina na segunda avaliação	0,528 (0,218–1,279)	0,142		
% de elevação da troponina em duas avaliações sequenciais	1,000 (0,999–1,001)	0,750		
Elevação isolada da troponina em duas avaliações sequenciais	0, 239 (0,111–0,512)	<0,001	0,433 (0,196–0,958)	0,039
Hospitalização no evento índice	3.782 (1.794–7.973)	<0.001	4.708 (1.652–13.423)	0.004

IC 95%: intervalo de confiança de 95%; DAC: doença arterial coronariana; IC: insuficiência cardíaca; TFG: taxa de filtração glomerular de acordo com a equação MDRD; CK: creatina quinase; CK-MB: creatina quinase-MB; Inibidor da ECA: inibidor da enzima conversora da angiotensina; ARM: antagonista do receptor mineralocorticoide; ECG: eletrocardiograma.

## Discussão

### Interpretação

Em nosso estudo, 78% dos pacientes com elevação da troponina receberam diagnóstico de não-SCA, o que está de acordo com estudos anteriores.^20^^,^^21^ Em algumas séries, a isquemia miocárdica não foi identificada em aproximadamente 65% dos pacientes do PS com elevação da troponina. O espectro do diagnóstico clínico foi extremamente heterogêneo em nosso estudo, englobando condições de alto risco. O prognóstico a médio prazo foi claramente pior para pacientes com troponina elevada e sem SCA do que para pacientes com níveis normais de troponina. Provavelmente também foi pior do que para pacientes com diagnóstico de SCA, com esses achados relatados em estudos anteriores.^16^^,^^22^^,^^23^

Neste estudo, 33,5% dos pacientes com níveis elevados de troponina e diagnóstico de não-SCA tiveram alta do PS sem internação. Esse percentual parece muito alto, mas taxas mais elevadas foram descritas por outros autores.^21^^,^^24^^,^^25^ No grupo sem SCA, a mortalidade em 16 meses foi de 38,6%, mas 81,4% dessas mortes ocorreram durante a internação ou nos primeiros 30 dias, o que reforça o papel do MNM como preditor de mortalidade nesse grupo. Considerando a alta taxa de alta hospitalar após uma internação no PS e o alto risco de mortalidade conferido pela elevação da troponina, não é surpreendente que os pacientes idosos com elevação da troponina que recebem um diagnóstico diferente de SCA e não são hospitalizados apresentem em um risco inaceitável de morte.

Acreditamos que a alta taxa de mortalidade durante a hospitalização e seguimento está intimamente relacionada à idade avançada e maiores comorbidades (insuficiência cardíaca anterior ou medicação antiplaquetária), conforme relatado por outros autores.^21^

O único biomarcador recomendado para uso no diagnóstico de SCA neste momento é a Tnc, devido à sua sensibilidade e precisão superiores.^2^^,^^3^ De fato, até 80% dos pacientes com IM isquêmico terá um nível elevado de troponina nas 2 a 3 horas após a chegada ao PS.^7^

Nosso estudo é notável por descobrir que a elevação isolada da TncI em duas análises consecutivas de MNMs é um preditor de sobrevida para pacientes com elevação da TncI e sem SCA, em comparação com a elevação de pelo menos dois MNMs (TncI e CK-MB e/ou mioglobina). Algumas particularidades das diferentes propriedades das moléculas de MNM poderiam explicar esse fato. A mioglobina apresenta liberação precoce e *clearance* rápida (liberada a partir de 1h após a lesão e retorna à linha de base em 24-36h), enquanto a CK-MB apresenta uma liberação e *clearance* mais lentas (liberada a partir de 4-9h após a lesão e *clearance* em 48-72h),^26^ e a troponina apresenta liberação semelhante à da CK-MB (4–9h), mas *clearance* retardada (7–10 dias).^27^ Nossa hipótese é que a elevação persistente da CK-MB e/ou mioglobina junto com a troponina em duas análises consecutivas de MNM implica em uma lesão miocárdica recente ou permanente, mesmo em pacientes sem SCA.

Provavelmente houve uma diferença no mecanismo de liberação de diferentes moléculas de MNM de acordo com o tipo e a gravidade da lesão. Alguns estudos em animais e células humanas sugeriram que a descarga de proteínas miocárdicas, exatamente como a Tnc, pode não implicar em necrose miocárdica.^28^

Em relação às subunidades T e I, a TncI tem um peso molecular de 37 kDa e a TncT tem um peso molecular de 21 kDa, ambas presentes principalmente nos sarcômeros e 4–6% no citoplasma. Após a lesão miocárdica, a troponina citosólica é liberada primeiro; à medida que mais danos ocorrem, a troponina presente no sarcômero é liberada na circulação;^8^ estudos anteriores defenderam a ideia de que cardiomiócitos com lesão reversível poderiam liberar troponina.^29^^–^^31^

A CK-MB também é liberada com necrose tecidual devido ao seu alto peso molecular (86kDa). A mioglobina tem liberação rápida, provavelmente pelo baixo peso molecular (17kDa) e sua localização citoplasmática, podendo ser liberada sob estresse miocárdico sem necrose,^8^ assim como a troponina.

Essa propriedade molecular de diferentes MNMs poderia explicar a incapacidade de uma medida isolada da troponina I em predizer mortalidade no presente estudo e em outro.^32^ Este achado destaca o papel valioso da CK-MB e da mioglobina que não pode ser realizado pela medida isolada da troponina. No entanto, as diretrizes atuais recomendam que a Tnc seja o único biomarcador utilizado para o diagnóstico da SCA, devido à sua sensibilidade e precisão superiores.^2^^,^^3^ No entanto, a não-realização das medidas de CK-MB e mioglobina pode ter um custo, especialmente para aqueles pacientes com um diagnóstico de não-SCA. Acreditamos que a exclusão da CK-MB e mioglobina da avaliação de rotina com MNMs em muitas instituições e diretrizes deve ser reconsiderada, devido ao seu valor prognóstico adjuvante superior, principalmente em pacientes sem SCA, e o aumento do número de pacientes com elevação da troponina que serão observados com níveis de troponinas altamente sensíveis.

### Limitações

Nossa instituição segue um protocolo não-restritivo para pedidos de medidas de MNM no PS. Portanto, nossa taxa de pacientes sem SCA estava provavelmente aumentado em comparação com protocolos mais rígidos.

Nosso estudo analisou a mortalidade dos pacientes sem considerar que os pacientes foram tratados de forma diferente de acordo com o diagnóstico inicial. Esta pode parecer uma limitação importante, mas deve ser esclarecido que cada processo clínico geralmente tem seu próprio manejo específico que influencia o prognóstico do paciente. Portanto, o prognóstico dos grupos é inerente, de alguma forma, ao manejo fornecido. Por exemplo, os pacientes com SCA são geralmente admitidos para tratamento com agentes antiplaquetários, anticoagulantes, estatinas, revascularização e outras terapias, e essa abordagem tem um prognóstico específico.

Os dados foram coletados retrospectivamente, sendo possível que alguns prontuários estivessem incompletos e o histórico clínico subvalorizado.

O ensaio de troponina utilizado anteriormente em nosso hospital era um ensaio contemporâneo denominado ‘troponina sensível’ e não era altamente sensível, ao contrário do ensaio de troponina utilizado atualmente, através do qual espera-se detectar valores positivos de troponina em mais pacientes, como descrito para este ensaio de troponina.^18^^,^^19^

## Conclusão

Uma alta porcentagem de pacientes com um nível elevado de troponina medido no PS não foi diagnosticada com SCA. Esses pacientes apresentavam perfil clínico de alto risco, ampla heterogeneidade em relação ao diagnóstico principal e prognóstico adverso aos 16 meses. Uma elevação isolada da troponina I em duas determinações consecutivas de MNMs foi um forte preditor de sobrevida em pacientes sem SCA com elevação da troponina.
