# Supplementary material for: Valor Prognóstico de Níveis Elevados de Troponina I Isolados em Pacientes sem Síndrome Coronariana Aguda Admitidos no Serviço de Emergência
Source: Arq Bras Cardiol. 2021 May 6;116(5):928–37. [Article in Portuguese] doi: 10.36660/abc.20190356 (PMC8121477; doi:10.36660/abc.20190356)
Supplement: Translation

# Translation: Prognostic Value of Isolated Elevated Troponin I Levels in Patients without Acute Coronary Syndrome Admitted to the Emergency Department

**Authors:** Célia Domingues, Maria João Vidigal Ferreira, Joana Moura Ferreira, Ana Vera Marinho, Patrícia Marques Alves, Cátia Ferreira, Isabel Fonseca, Lino Gonçalves

**Affiliations:** 1 Coimbra Portugal Centro Hospitalar e Universitário de Coimbra EPE, Coimbra - Portugal; 2 Coimbra Portugal Universidade de Coimbra - Faculdade de Medicina, Coimbra - Portugal

**Keywords:** Troponin I, Prognosis, Emergency Department, Myocardial Non-Ischemic Injury

## Abstract

**Background:**

Although non-ischemic troponin elevation is frequently seen in patients admitted to the emergency department (ED), consensus regarding its management is lacking.

**Objectives:**

This study aimed to characterize patients admitted to the ED with non-ischemic troponin elevation and to identify potential mortality predictors in this population.

**Methods:**

This retrospective observational study included ED patients with a positive troponin test result between June and July of 2015. Patients with a clinical diagnosis of acute coronary syndrome (ACS) were excluded. Data on patient demographics and clinical and laboratory variables were extracted from medical records. Follow-up data were obtained for 16 months or until death occurred. The statistical significance level was 5%.

**Results:**

Troponin elevation without ACS was found in 153 ED patients. The median (IQR) patient age was 78 (19) years, 80 (52.3%) were female and 59(38.6%) died during follow-up. The median (IQR) follow-up period was 477(316) days. Survivors were significantly younger 76 (24) vs. 84 (13) years; p=0.004) and featured a higher proportion of isolated troponin elevation (without creatine kinase or myoglobin elevation) in two consecutive evaluations: 48 (53.9%) vs. 8 (17.4%), p<0.001. Survivors also presented a lower rate of antiplatelet treatment and same-day hospitalization. In the multivariate logistic regression with adjustment for significant variables in the univariate analysis, isolated troponin elevation in two consecutive evaluations showed a hazard ratio= 0.43 (95%CI 0.17–0.96, p=0.039); hospitalization, previous antiplatelet treatment and age remained independently associated with mortality.

**Conclusions:**

Isolated troponin elevation in two consecutive measurements was a strong predictor of survival in ED patients with troponin elevation but without ACS.

## Introduction

Clinical myocardial infarction according to the fourth universal definition requires the presence of acute myocardial injury detected by abnormal cardiac biomarkers associated with evidence of acute myocardial ischemia. Cardiac troponin(cTn) above the 99^th^ percentile cutoff point with an increasing or decreasing pattern is the biomarker of myocardial injury,^1^ because it cannot be released by non-cardiac tissues and has an excellent accuracy for the diagnosis of acute myocardial infarction.^1^^–^^3^

cTn is a protein distributed within the cytoplasm and sarcomere of a cardiac myocyte, mostly in the sarcoplasmic reticulum. Three subunits make up the troponin complex, an inhibitory component (troponin I), tropomyosin-binding component (troponin T) and calcium-binding component (troponin C).^4^

The T and I subunits (cTnT and cTnI, respectively) are specific to cardiac muscle and, thus, can act as suitable markers of cardiac injury. cTnT shows a double discharge, first the cytoplasmic component and later the binding component.^5^ cTnI is cardiac-specific, and was not identified in skeletal muscle. This 100% specificity shows cTnI can be an ideal myocardial necrosis marker (MNM).^6^

Before the advent of troponin, the previous MNM used were muscle/brain isoenzyme of creatine kinase (CK-MB) and myoglobin, which were less sensitive and not specific to myocardial infarction.^7^^,^^8^ Due to their lack of sensitivity and specificity, they have been progressively excluded from ACS investigations.^2^^,^^3^

Although cTn subunits are strongly specific to cardiac myocytes, they can be released under a wide spectrum of non-cardiac pathological conditions, such sepsis, chronic kidney disease, hypertensive emergencies, gastrointestinal bleeding, stroke and rhabdomyolysis.^6^^,^^9^ In this setting, the detection of troponin may be the result of 5–8% of the cytosolic component release in response to myocyte cell turnover, cellular release of degradation products, and increased cellular wall permeability.^10^

The widespread use of troponin assays in the emergency department (ED) may pose a difficult diagnostic challenge when the test is abnormal in patient without an ACS.^11^According to the literature, elevated cTn levels in patients without an ACS was associated with a poor prognosis.^10^^,^^12^^–^^16^

## Objective

This study aimed to identify factors predictive of mortality/survival in ED patients without ACS and elevated cTnI using patient characteristics, clinical past history, comorbidities and analytic values (including creatinine, CK-MB, myoglobin and cTnI) measured in ED.

## Methods

### Study design, setting, and participants

In this retrospective study, we analyzed the laboratory data of consecutive patients that came to the ED of a community teaching hospital during 1-month period from June to July of 2015, and selected patients who presented with cTnI elevation.

All clinical information was collected, including medical and nursing records of the ED and hospitalizations, analyses, and other complementary exams. Follow-up was performed through local and national records to observe the rates of cardiovascular re-hospitalization and death.

The collected data included demographics, cardiovascular risk factors, myocardial biomarker assay and creatinine results, main symptom at the ED, final ED diagnosis, hospitalization, hospitalization mortality, mortality rate at 30 days and 16 months, and cardiovascular re-hospitalization rate.

In this study, the criteria for the diagnosis of acute myocardial infarction were an increase or decrease in Troponin I with at least one abnormal value higher than the upper reference limit of the assay and at least one of the following: 1) symptoms of ischemia; 2) new ST-segment/ T-wave changes or new left bundle-branch block; 3) development of pathological Q waves on electrocardiography; 4) new loss of viable myocardium or regional wall motion abnormalities on imaging test; or 5) identification of an intracoronary thrombus on imaging test.^1^

Emergency and hospitalization registries of all patients with elevated cTnI, were revised by the investigators, and patients were divided into two groups: those diagnosed with an ACS, (Type 1 myocardial infarction(MI) or type 2 with ischemic signs and symptoms (with vasospasm, embolism and non-atherosclerotic coronary dissection) (group A) and those without an ACS with a positive troponin assay due to oxygen supply/demand imbalance or myocardial injury with no signs or symptoms of acute myocardial ischemia(group B). Patients with type 4 or 5 MI were not included in this study, as by definition these are not the regular ED patients, and type 3 MI patients have no cTnI measure.^1^

Non-ACS patients were identified by predefined criteria that included the following: 1) myocarditis/cardiomyopathy: discharge diagnosis or findings suggestive of myocarditis on imaging test or pathology, infiltrative cardiomyopathies such as amyloidosis or sarcoidosis, an ejection fraction ≤30% prior to admission, or prior cardiac transplantation; 2) infections: conditions with systemic impact such as cellulitis, pneumonia, sepsis, and pyelonephritis; 3) acute dysrhythmias unrelated to ACS; 4) chronic or acute kidney disease: stage-5 chronic kidney disease, chronic dialysis, renal transplant recipient, or moderate to severe acute kidney disease; 5) central nervous system pathology: stroke, seizure, or subarachnoid hemorrhage; 6) acute abdominal or gastrointestinal bleeding; 7) pulmonary embolism; 8) unexplained syncope; 9) asthma or chronic obstructive pulmonary disease exacerbation; and 10) others: elevated troponin level of unknown etiology, which does not meet any of the abovementioned criteria.

The primary endpoint for this study was mortality during hospitalization, at 30 days and 16 months, while the secondary endpoint was rehospitalization for cardiovascular disease during follow-up.

Follow-up was completed at 16 months or at death. Follow-up was performed through electronic medical records and the online national death registry. The institutional review board approved the study protocol. The requirement for informed consent was waived, because the patients did not receive any type of different care because of the study.

### Myocardial necrosis marker assays

The troponin I level was determined using the same standard cTnI immunoassay (Troponin I Siemens Dimension EXL)^17^^–^^19^ in all patients. The test was performed in the central hospital laboratory. The lower and upper detection limits established by the manufacturer were 0.017 ng/mL and 4000ng/mL, respectively. Measurements below the detection limit were assigned a value of 0. Troponin I test results were considered positive if the level was higher than the reference limit (>0.059 ng/mL) used in the ED laboratory. The CK-MB and myoglobin assay results were considered normal at <3.6ng/mL and at 9–82 ng/mL, respectively.

Repeated MNM measurements were performed at least 3 hours after the first evaluation.

Using the first and second evaluation of cardiac troponin, the variation was calculated as follows: variation of troponin % = ((second troponin × 100)/first troponin) × 100%.

### Statistical methods

All continuous data were tested for normality with the Shapiro-Wilks test; all showed non-normal distribution and are presented as median and interquartile range. The Mann-Whitney test was applied to compare continuous variables. Categorical variables were represented by their frequency and compared using Fisher's exact test or the chi-square-test.

Survival was analyzed using uni- and multivariate Cox proportional hazard models. The results were expressed as hazard ratio (HR) with 95% confidence intervals (95%CI). For the independent survival predictor variables, a survival plot was obtained using the Kaplan-Meier method and the log-rank test. The statistical significance level was set at p-value<0.05. All statistical analyses were performed using SPSS 23.0 for Mac (SPSS, Inc; Chicago, IL, USA).

## Results

### Baseline characteristics and diagnoses of the study population

During the one-month study period, 10,564 patients were admitted to our institution's ED. Patients who underwent MNM evaluations including troponin I, CK-MB, and myoglobin and their distribution according to troponin status and final diagnosis are described in Figure 1. Patients were assigned to two groups: Group A (n=42 [21.5%]) with ACS (all of them with acute occlusion/subocclusion of coronary arteries: 4 of them with type 2 MI (2 coronary dissection and 2 embolic coronary thrombosis); of 38 with type 1 MI, 14 with acute MI with ST-elevation (STEMI)); while in Group B (n=153 [78.5%]) with Non-ACS, 58 had oxygen supply/demand imbalance, 53 had acute myocardial injury without sign or symptoms of ischemia and 42 had stable elevation of cTnI (cTnI variation on two consecutive analysis ≤ 20%). Among the Non-ACS patients, the first measure of MNM was performed after a median of 6 (IQR 4) hours since symptom onset, and 90 patients had the MNM measure repeated after a median of 5 (IQR 3) hours since the first evaluation.

**Figure 1:** Schematic illustration of patients included. MNM, myocardial necrosis markers; ACS, acute coronary syndrome

On the first MNM evaluation of group B, 81 had CKMB and/or myoglobin elevation (16 showed CKMB and 40 showed myoglobin elevation, whereas 25 showed elevation of both). On the second evaluation, 18 showed elevation of both and 6 showed isolated elevation of CKMB and 31 of myoglobin. At both evaluations, 88 patients showed at least one elevation of CKMB and/or myoglobin.

Patients with a positive troponin test result had a wide spectrum of clinical symptoms at presentation (Figure 2). As expected, patients who ultimately received a diagnosis of ACS (Group A) had a higher proportion of chest pain as the main complaint at hospital presentation.

**Figure 2:** Schematic illustration of clinical symptoms at presentation of all patients and different groups A: with ACS and group B: Non ACS.

As shown on Table 1, patients in Groups A and B had a similar median age but had significantly different gender proportions. Concerning cardiovascular risk factors and comorbidity conditions, no significant differences were found in the prevalence of diabetes mellitus and hypertension, but hyperlipidemia and previous coronary artery disease were more common in the ACS patients and previous heart failure and anticoagulant treatment were more prevalent in Non-ACS patients.

**Table 1:** Baseline characteristics of all patients with troponin elevation at the ED

	Overall N = 195	Group A n = 42 (22%)	Group B n = 153 (78%)	p Value
Age (years), median(IQR)	77(21) years	71(19) years	78(19) years	0.06
Male n (%)	105 (53.8%)	32 (76.2%)	73 (47.7%)	0.001
Diabetes mellitus, n (%)	69 (35.5%)	16 (37.2%)	54 (35%)	0.89
Hypertension, n (%)	155 (79.3%)	35 (84.4%)	119 (78.1%)	0.57
Hyperlipidemia, n (%)	87 (44.4%)	25 (60%)	61 (40.1%)	0.03
Previous CAD, n (%)	37 (19%)	13 (31%)	24 (16%)	0.02
Previous HF, n (%)	43 (21.9%)	9 (21.9%)	71 (46.7%)	0.02
Previous AIS, n (%)	31 (14.8%)	3 (6.3%)	26 (16.8%)	0.26
GFR (ml/[min⋅1.73 m^2^]), median(IQR)	54 (46)	68 (47)	49(47)	0.10
**Previous Medications**				
Anticoagulants	62 (33.7%)	4 (10%)	47 (31%)	0.007
Antiplatelets	50 (26.7%)	15(37%)	48 (32%)	0.13
Beta-blockers	69 (36.9%)	16 (40%)	53 (36.1%)	0.65
ACE inhibitors/AARA	108 (57.8%)	23 (57.5%)	87 (57.8%)	0.97
MRA	15 (17.6%)	2 (12.5%)	13 (18.8%)	0.55
Statins	83 (44.4%)	24 (60%)	60 (40.1%)	0.02
Standardized troponin at the first evaluation*, n (%)				<0.001
1–2.99	95 (48.9%)	10 (23.1%)	85 (55.8%)	
3–4.99	36 (18.3%)	5 (12.8%)	31 (19.7%)	
5–9.99	19 (9.7%)	4 (10.3%)	15 (9.7%)	
10+	45 (23.1%)	23 (53.8%)	22 (15%)	
Elevated CK-MB, n(%)	54 (53%)	22 (53%)	32 (21%)	<0,001
Elevated myoglobin, n(%)	88 (48%)	23 (56%)	66 (45%)	0,24
Elevated CK-MB + Myoglobin, n(%)	43 (22%)	16 (40%)	25 (16%)	0,003
% troponin elevation between 2 measurements, median (IQR)	7 (73)	183 (666)	2,65(42)	<0.001

Group A- patients with acute coronary syndrome (ACS); Group B- patients without ACS. CAD: coronary artery disease; HF: heart failure; AIS: acute ischemic stroke; GFR: glomerular filtration rate according to the MDRD equation; ACE inhibitor: angiotensin-converting enzyme inhibitor; MRA: mineralocorticoid receptor antagonist.

The main diagnoses in Group B patients were myocarditis/cardiomyopathy (40[26%]), followed by infection (cellulitis, pneumonia, and pyelonephritis, 24[15.5%]), acute dysrhythmias (25 [16.6%]), chronic or acute kidney disease (17 [11%]), cerebral disease (13 [8.4%]), acute abdominal or gastrointestinal bleeding (11 [7.1%]), pulmonary embolism (6 [3.9%]), unexplained syncope (4[2.6%]), asthma or chronic obstructive pulmonary disease exacerbation (4 [2.6%]), and others (9 [6.5%]).

### Outcome data

The median (IQR) follow-up was 477 days (316). No significant intergroup differences were found regarding in-hospital mortality (6 [14.3%] vs. 21 [13.7%], p=0.077), 30-day mortality (6 [14.3%] vs. 27 [17.6%], p=0.4) and cardiovascular rehospitalization at follow-up (11 [29.7%] vs 32 [24.2%], p=0.316). Remarkably, the long-term mortality rate was significantly higher in group B patients (9 [21.4%] vs. 59 [38.6%], p=0.039), although the survival curves of the two groups were not significantly different (log rank, 3.45; p=0.063). The main causes of death in group B were cardiovascular in 12 individuals (none of them with a diagnosis of acute myocardial infarction), non-cardiovascular in 27, 13 unknown causes and 4 due to mixed of cardiovascular and other comorbidity.

### Main results

Predictors of mortality in Group B patients are described in Table 2. We found that older age (p<0.001), previous heart failure (p=0.049), previous antiplatelet medication (p=0.005) and hospitalization after index ED evaluation (p<0.001) were mortality predictors. Death at follow-up was not related to cTnI, CK-MB, or myoglobin levels (first, second, or both evaluations). However, isolated troponin elevation (i.e., without concomitant CK-MB or myoglobin elevation) was a powerful predictor of survival; in fact, isolated troponin elevation at the first measurement was present in 58.7% of survivors vs. 40% of non-survivors (p=0.021); patients with two isolated troponin elevation measurements were more likely to survive (48 [53.9%] of survivors vs. 8[17.4%] of non-survivors [p<0.001]).

**Table 2:** Association between Clinical Variables and Long-Term Survival of Patients with Troponin Elevation and Non-Acute Coronary Syndrome (Group B)

	Survivors (n = 94)	Non-survivors (n = 59)	p-value
Age, median (IQR)	76(24) years	84 (13)	<0.001
Male, n(%)	44 (46.8%)	29 (49.2%)	0.77
**CV Risk Factors, n(%)**			
Diabetes mellitus	28 (30.8%)	22 (38.6%)	0.33
Hypertension	68 (73.9%)	44 (78.6%)	0.52
Previous CAD	14 (15.2%)	9 (16.4%)	0.85
Previous HF	33 (39.3%)	31 (56.4%)	0.049
GFR, mL/(min⋅1.73 m^2^), median(IQR)	56 (48)	45 (34)	0,05
Heart rate, bpm, median(IQR)	75 (33)	84 (36)	0.10
**Previous Medication, n (%)**			
Antiplatelets	22 (23.9%)	25 (35.5%)	0.02
Anticoagulants	34 (37%)	12 (21.8%)	0.06
Beta-blockers	35 (38%)	18 (32.7%)	0.52
ACE inhibitors	57 (62%)	28 (50.9%)	0.19
MRA	9 (20.5%)	4 (16%)	0.65
Statins	36 (39.1%)	23 (41.8%)	0.75
**ECG, n (%)** No significant alterations	51 (64.6%)	16 (50%)	0.38
ST elevation	1 (1.3%)	0 (0%)	
ST-depression or negative T-wave	16 (20.3%)	6 (18.8%)	
Atrial fibrillation	28 (35.0%)	18 (46.2%)	
LBBB	4 (5.1%)	4 (12.5%)	
Pace rhythm	4 (5.1%)	3 (9.4%)	
**Myocardial Necrosis Markers, median (IQR)**			
Troponin (ng/mL) at the first evaluation	0,13 (0,23)	0,10 (0,18)	0.61
CK-MB (ng/mL) at the first evaluation	1,6 (1,8)	1,9 (2,05)	0.50
Myoglobin (ng/mL) at the first evaluation	70 (120)	99 (175)	0.06
Isolated troponin elevation at the first evaluation, n (%)	54 (58.7%)	22 (40%)	0.028
Troponin (ng/mL) at the second evaluation, median (IQR)	0,12(0,16)	0,14(0,32)	0,28
% troponin I variation on two sequential measurements, median (IQR)	0 (32)	27 (35)	0.002
Isolated troponin elevation at two sequential measurements, n(%)	48 (53.9%)	8 (17.4%)	<0.001
Hospitalization at index event, n(%)	52 (55.3%)	51 (86.4%)	<0.001
Coronary revascularization			0.88
No specific therapy, n(%)	88 (93.6%)	54 (91.5%)	
OMT, n(%)	5 (5.3%)	4 (6.8%)	
PCI + OMT, n(%)	1 (1.1%)	1 (1.1%)	

IQR: interquartile range; CAD: coronary artery disease; HF: heart failure; AIS: acute ischemic stroke; GFR: glomerular filtration rate according to MDRD equation; ACE inhibitor: angiotensin-converting enzyme inhibitor; MRA: mineralocorticoid receptor antagonist; ECG: electrocardiogram; CK: creatine kinase; CK-MB: creatine kinase-MB; OMT: optimized medical therapy; PCI: percutaneous coronary intervention.

Cox regression analysis is shown on Table 3. The univariate analysis showed that in inpatients with isolated troponin in two consecutive MNM measurements, the probability of long-term survival at follow-up increased by four-fold (p<0.001). Cox multivariate analysis corrected for age and gender demonstrated that isolated troponin elevation in two consecutive measurements remained an independent predictor of survival (HR, 0.433; 95%CI, 0.196–0.958; p=0.039). Figure 3 shows the survival curves of Group B patients according to the presence of isolated troponin elevation in two consecutive measurements (log rank, 18.09; p<0.001).

**Table 3:** Univariate and multivariate (corrected for age and gender) Cox regression analysis of clinical variables and long-term survival of patients with troponin elevation and non-acute coronary syndrome

	Univariate Cox regression		Multivariate Cox Regression	
	HR (95% CI)	p Value	HR (95% CI)	p Value
Age, y	1.040 (1.017–1.063)	0.001	1,030 (1,002–1,058)	0.038
Gender	0.899 (0.540–1.499)	0.684	0,807 (0,436–1,493)	0.494
CV risk factors				
Diabetes mellitus	1.336 (0.783–2.277)	0.288		
Hypertension	1.245 (0.658–2.358)	0.501		
Previous CAD	1.067 (0.521–2.182)	0.860		
Previous HF	1.649 (0.967–2.812)	0.066		
GFR, mL/(min⋅1.73 m^2^)	0.992 (0.984–1.001)	0.082		
Heart rate, bpm	1.006 (0.997–1.014)	0.193		
**Previous Medication**				
Antiplatelets	1.867 (1.230–2.835)	**0.006**	1,823 (1,105–3,006)	**0.019**
Beta-blockers	0.806 (0.459–1.416)	0.449		
ACE inhibitors	0.689 (0.406–1.170)	0.170		
MRA	0.764 (0.262–2.226)	0.611		
Statins	1.017 (0.595–1.739)	0.950		
ECG pattern	1.162 (0.999–1.351)	0.067		
**Laboratory**				
Isolated troponin elevation at the first evaluation	0.533 (0.311–0.916)	**0.021**	1.097 (0.378–3.180)	0.865
Isolated troponin elevation at the second evaluation	0.528 (0.218–1.279)	0.142		
% troponin elevation at two sequential evaluations	1.000 (0.999–1.001)	0.750		
Isolated troponin elevation at two sequential evaluations	0. 239 (0.111–0.512)	**<0.001**	0.433 (0.196–0.958)	**0.039**
Hospitalization at index event	3.782 (1.794–7.973)	**<0.001**	4.708 (1.652–13.423)	**0.004**

95% CI: 95% confidence interval; CAD: coronary artery disease; HF: Heart failure; GFR: glomerular filtration rate according to MDRD equation; CK: creatine kinase; CK-MB: creatine kinase-MB; ACE inhibitor: angiotensin converting enzyme inhibitor; MRA: mineralocorticoid receptor antagonist; ECG: electrocardiogram.

**Figure 3:** Survival Kaplan Meier curve at 16-month follow-up of Non-ACS patients according to the results of two consecutive MNM evaluations.

## Discussion

### Interpretation

In our study, 78% of patients with troponin elevations received a non-ACS diagnosis, which is in agreement with previous studies.^20^^,^^21^ In some series, myocardial ischemia was not identified in approximately 65% of ED patients with troponin elevation. The spectrum of clinical diagnosis was extremely heterogeneous in our study, encompassing high-risk conditions. Mid-term prognosis was clearly worse for patients with elevated troponin and non-ACS than for patients with normal troponin levels. It was also probably worse than for patients diagnosed with ACS, with these findings having been reported in previous studies.^16^^,^^22^^,^^23^

In this study, 33.5% of the patients with elevated troponin levels and a non-ACS diagnosis were discharged from the ED without hospitalization. This percentage seems very high, but higher rates have been described by other authors.^21^^,^^24^^,^^25^ In the non-ACS group, the 16-month mortality rate was 38.6%, but 81.4% of these deaths occurred during hospitalization or during the first 30 days, which reinforces the role of MNM as a mortality predictor in this group. Considering the high hospital discharge rate after an ED stay and the high risk of mortality conferred by troponin elevation, it is not surprising that elderly patients with troponin elevation who receive a diagnosis other than ACS and are not admitted to the hospital are at an unacceptable high risk of death.

We believe that the high mortality rate during hospitalization and follow-up is closely related to older age and greater comorbidities (past heart failure or antiplatelet medication), as reported by others.^21^

The only biomarker recommended to diagnose ACS at this time is cardiac troponin, due to its higher sensitivity and accuracy.^2^^,^^3^ In fact, up to 80% of patients with ischemic myocardial infarction will have an elevated troponin level within 2–3 hours after ED arrival.^7^

Our study is remarkable for finding that isolated cTnI elevation in two consecutive analyses of MNMs is a survival predictor for patients with cTnI elevation and non-ACS, compared to the elevation in at least two MNMs (cTnI and CK-MB and/or myoglobin). Some particularities of the different properties of MNM molecules could explain this fact. Myoglobin has an early release and quick clearance (released starting 1h after the injury and return to baseline at 24–36h), while CK-MB shows slower release and clearance (release starting 4–9h after the injury and clearance after 48–72h),^26^ and troponin shows a release similar to that of CK-MB (4–9h) but delayed clearance (7–10 days).^27^ We hypothesize that the persistent elevation of CK-MB and/or myoglobin along with troponin in two consecutive MNM analyses implies a recent or permanent myocardial injury, even in non-ACS patients.

Probably, there was a difference in the release mechanism of different MNM molecules according to the injury type and severity. Some studies in animals and human cells suggested that the discharge of myocardial proteins just like cTn may not imply myocardial necrosis.^28^

Regarding the T and I subunits, cTnI has a molecular weight of 37 kDa and cTnT has a molecular weight of 21 kDa; both presenting mainly in the sarcomeres and 4–6% in the cytoplasm. After the myocardial injury, cytosolic troponin is released first; as further damage occurs, the troponin present in the sarcomere is released into the circulation;^8^ previous studies advocated that reversibly injured cardiomyocytes could release troponin.^29^^–^^31^

CK-MB is also released when there is tissue necrosis, because of its high molecular weight (86kDa). Myoglobin has a rapid release, probably because of its low molecular weight (17kDa) and cytoplasmic location, and it could be released under myocardial stress without necrosis,^8^ just like troponin.

This molecular property of different MNMs could explain the incapacity of a single troponin I measurement to predict mortality in the present study and another one.^32^ This finding highlights the valuable role of CK-MB and myoglobin that cannot be accomplished by an isolated troponin measurement. However, current guidelines recommend that cardiac troponin be the only biomarker used for the diagnosis of ACS, owing to its superior sensitivity and accuracy.^2^^,^^3^ Nevertheless, failing to perform CK-MB and myoglobin measurements may come at a cost, especially for those patients who receive a non-ACS diagnosis. We believe that the exclusion of CK-MB and myoglobin from routine MNM evaluations in many institutions and guidelines should be reconsidered because of its superior adjunctive prognostic value, mainly in non-ACS patients, and the increased number of patients with troponin elevation that will be observed with highly sensitive troponin levels.

### Limitations

Our institution follows a non-restrictive protocol for MNM measurement orders at the ED. Therefore, our rate of Non-ACS probably were increased, when compared with more strict protocols.

Our study analyzed patient mortality without considering that patients were managed differently according to the initial diagnosis. This may seem to be an important limitation, but it should be clarified that each clinical process usually has its own specific management, which influences patient prognosis. Therefore, the prognosis of the groups is somehow inherent to the provided management. For instance, patients with ACS are usually admitted for treatment with antiplatelet, anticoagulant agents, statins, revascularization, and other therapies, and this approach has a specific prognosis.

Data were retrospectively collected and it is possible that some medical records were incomplete and the clinical history could have been undervalued.

The troponin assay previously used in our hospital was a contemporary assay called ‘sensitive troponin’ and it was not highly sensitive, unlike the currently used troponin assay, which is expected to detect positive troponin values in more patients, as described for this troponin assay.^18^^,^^19^

## Conclusion

High percentage of patients with an elevated troponin level measured in the ED were not diagnosed with ACS. These patients had a high-risk clinical profile, broad heterogeneity regarding the main diagnosis, and an adverse prognosis at 16 months. An isolated troponin I elevation in two consecutive determinations of MNM was a strong predictor of survival in non-ACS patients with troponin elevation.
